# Probiotic Mixture Golden Bifido Prevents Neonatal *Escherichia coli* K1 Translocation via Enhancing Intestinal Defense

**DOI:** 10.3389/fmicb.2017.01798

**Published:** 2017-09-20

**Authors:** Qing Zeng, Xiaolong He, Santhosh Puthiyakunnon, Hansen Xiao, Zelong Gong, Swapna Boddu, Lecheng Chen, Huiwen Tian, Sheng-He Huang, Hong Cao

**Affiliations:** ^1^Department of Microbiology, Guangdong Provincial Key Laboratory of Tropical Disease Research, School of Public Health, Southern Medical University Guangzhou, China; ^2^The First School of Clinical Medicine, Southern Medical University Guangzhou, China; ^3^Saban Research Institute, Children’s Hospital Los Angeles, University of Southern California, Los Angeles CA, United States

**Keywords:** probiotics, intestinal barrier, bacterial translocation, neonatal sepsis and meningitis, mucin, tight junction

## Abstract

*Escherichia coli* (*E. coli*) K1 sepsis and meningitis is a severe infection characterized by high mortality in neonates. Successful colonization and translocation across the intestinal mucosa have been regarded as the critical steps for *E. coli* K1 sepsis and meningitis. We recently reported that the probiotic mixture, Golden Bifido (containing live *Lactobacillus bulgaricus, Bifidobacterium*, and *Streptococcus thermophilus*, LBS) has a preventive role against neonatal *E. coli* K1 bacteremia and meningitis. However, the interaction between the neonatal gut barrier, probiotics and *E. coli* K1 is still not elucidated. The present study aims to investigate how LBS exerts its protective effects on neonatal gut barrier during *E. coli* K1 infection. The beneficial effects of LBS were explored *in vitro* and *in vivo* using human colon carcinoma cell lines HT-29 and rat model of neonatal *E. coli* K1 infection, respectively. Our results showed that stimulation with *E. coli* K1 was able to cause intestinal barrier dysfunction, which were reflected by *E. coli* K1-induced intestinal damage and apoptosis of intestinal epithelial cells, reduction of mucin, immunoglobulin A (IgA) and tight junction proteins expression, as well as increase in intestinal permeability, all these changes facilitate *E. coli* K1 intestinal translocation. However, these changes were alleviated when HT-29 cells were treated with LBS before *E. coli* K1 infection. Furthermore, we found that LBS-treated neonatal rats (without *E. coli* K1 infection) have showed higher production of mucin, ZO-1, IgA, Ki67 in intestinal mucosa as well as lower intestinal permeability than that of non-treated rats, indicating that LBS could accelerate the development of neonatal intestinal defense. Taken together, our results suggest that enhancement of the neonatal intestinal defense to fight against *E. coli* K1 translocation could be the potential mechanism to elucidate how LBS confers a protective effect against neonatal *E. coli* K1 bacteremia and meningitis. This indirect mechanism makes LBS exert preventive effect on most of gut-derived pathogenic infections rather than only *E. coli*.

## Introduction

Bacterial sepsis and meningitis are regarded as severe infections that lead to high morbidity and mortality during the neonatal period (Bhagat et al., 2015). Among the bacterial pathogens causing sepsis and meningitis, the most common is *Escherichia coli* (*E. coli*) K1 (Glode et al., 1977). During the past decades, antibiotic resistance has become a major health issue affecting public health (Furuya and Lowy, 2006). The excessive, even indiscriminate use of antibiotics may increase the incidence of neonatal infections with antibiotic-resistant *E. coli* as well as reduces the efficacy of conventional antibiotic chemotherapy. Currently, the incidence of early onset *E. coli* infections and ampicillin-resistant *E. coli* infections has been increased rapidly in low birth weight infants (Simonsen et al., 2014). Given the seriousness of neonatal *E. coli* K1 infection, development of a non-antibiotic strategy for prevention and treatment has emerged as an urgent issue.

Bacterial translocation refers to migration of bacteria or bacterial products from the intestinal lumen through its epithelium to the extra-intestinal sites, such as the mesenteric lymph nodes and the blood stream (Koutsounas et al., 2015). It has already been established that vertical transmission is the primary route of neonatal *E. coli* K1 infection, which is usually spread from the maternal birth canal to fetal oral cavity at the time of birth (Kim, 2003; Witcomb et al., 2015). Following initial colonization in the intestinal mucosa, *E. coli* K1 could penetrate the intestinal mucosal barrier and enter into the blood stream resulting in bacteremia and sepsis (Yousuf et al., 2014). Once reaches a necessary threshold in blood, *E. coli* K1 penetration across the blood brain barrier and invade the central nervous system, leading to meningitis (Wang et al., 2016). Obviously, the pathogenesis of *E. coli* K1 is a multi-step process, while successful translocation across the gut barrier could be a crucial step for meningeal invasion by *E coli* K1. Although the precise mechanism of neonatal *E. coli* K1 translocation is not completely understood, it is clear that this complicated process would occur as a result of interactions between the enteropathogenecity of *E. coli* K1 and the immaturity of the neonatal intestinal defense.

Gut barrier is a multi-layer system consisting of two functional units: physical barrier and functional barrier. The intestinal physical barrier is the first line of defense against pathogens, and it plays an important role in maintaining the segregation between host and intestinal microbiota. From the outer layer to the inner layer, intestinal physical barrier is composed of commensal microbiota, mucus layer, intestinal epithelial cells layer and the epithelial tight junction. Commensal microbiota could compete for adhesion sites and nutritional resource with pathogens, and maintain intestinal homeostasis (Smith et al., 2007; Yang et al., 2015) as well as promote the maturation of the intestinal immune system. Intestinal mucus layer is a dense layer which lie on the surface of intestinal epithelium, contains antimicrobial products and secretory immunoglobulin A (IgA), that prevents bacterial penetration (Hansson, 2012; Ouwerkerk et al., 2013). The last but not the least, the intestinal epithelial layer, which consist of enterocytes, goblet cells, endocrine cells, paneth cells, microfold cells etc., are joined together by tight junctions and form a contiguous and relatively impermeable barrier, inhibiting the translocation of bacteria to inner tissues (Bruewer et al., 2006; Naydenov and Ivanov, 2010; Blume et al., 2016). Below the intestinal physical barrier is the immune barrier, consisting of aggregated lymphoid follicles and scattered immune cells (Constantinovits et al., 2012; Pearson et al., 2012). Many studies have suggested that *E. coli* K1 infection could induce disruption of the gut physical barrier, and thereby translate across the intestinal lumen. For example, Valeri et al. (2015) found that *E. coli* K1 could express SsiE, a zinc-metalloprotease, which is associated with the cleavage of mucin and facilitates *E. coli* adhesion to intestinal epithelium. Burns et al. (2001) demonstrated that *E. coli* K1 could decrease the transepithelial cell electrical resistance of the intestinal barrier *in vitro*, indicating that *E. coli* K1 has potential to break the integrity of the intestinal barrier. Furthermore, Birchenough et al. (2013) showed that neonatal colonization of rats with neuropathogenic *E. coli* O18:K1 impaired the intestinal barrier function, including down-regulation of intestinal defensins, mucin and trefoil factor family 2 synthesis. Overall, these studies suggested that *E. coli* K1 could exert a deleterious effect on gut barrier function.

Poor intestinal defense is another pivotal reason responsible for neonatal susceptibility to *E. coli* K1 translocation. Recently, Birchenough et al. (2013) found that 9 days old wistar rat pups were more resistance to gut-originated *E. coli* K1 infection than 2 days old pups, as 9 days old pups have more sophisticated intestinal defense than 2 days old pups. The same phenomenon occurs in humans, because human neonates are also most susceptible to *E. coli* K1 infection during the early neonatal period (Holt et al., 2001). Now, it has been found that compared with adult, the intestinal defense of neonate, especially premature and very low birth weight infant, is relatively highly immature, which characterized as follows: (1) an immature intestinal immune system with a few T and B cell as well as a low-level of IgA (Claud, 2009); (2) a thin and sparse intestinal mucus layer (Birchenough et al., 2013); (3) a low diversity in gut flora mainly composed by aerobic bacteria, which is incapable of inhibiting pathogenic colonization and translocation (Madan et al., 2012); (4) low tight junction proteins expression and high intestinal barrier permeability (Weaver et al., 1984; Patel et al., 2012).

Currently, more evidences have demonstrated that probiotics exhibit the potential to maintain the intestinal homeostasis and prevent the bacterial translocation via enhancing intestinal barrier function (Zareie et al., 2006; Fukuda et al., 2011). Therefore, it is reasonable to predict that the application of probiotics in neonates would be a very promising strategy for preventing or treating neonatal *E. coli* K1 infection. Indeed, our recent research showed that a probiotic mixture Golden Bifido (contain live *Lactobacillus bulgaricus, Bifidobacterium*, and *Streptococcus thermophilus*, LBS) could exert a preventive effect on neonatal rats against gut-derived *E. coli* K1 infection (Yu et al., 2015). The present study aims at exploring how LBS exert its protective effect. Our data suggested that accelerating the development of neonatal intestinal defense and protecting intestinal barrier from *E. coli* K1’s enteropathogenecity could be the underlying mechanism to explain the contribution of LBS to neonatal resistance against *E. coli* K1 infection.

## Materials and Methods

### Ethics Statement

All animal experiments were approved by the Animal Care Committee of Southern Medical University (Guangzhou, China). Timed-pregnant Sprague-Dawley rats were obtained from Animal Experimental Center of Southern Medical University and bred in-house. All supplements including food, water, and other nutrients were autoclaved, and animals were kept in the animal facility. The protocol was approved by the Animal Care Committee of Southern Medical University. All surgeries were performed under anesthesia with ketamine and lidocaine, and utmost efforts were taken to minimize suffering.

### Bacterial Strains, Cell Lines and Culture Conditions

The probiotics used in this study was obtained from Live Combined *bifidobacterium* and *lactobacillus* Tablets [Trade Name: Golden Bifid, Inner Mongolia Shuangqi Pharmaceutical Co. Ltd., 0.5 g/tablet, no less than 50 million live *Bifidobacterium* (ATCC 15697), five million live *L. bulgaricus* (ATCC 11842) and *S. thermophilus* (ATCC 19987)]. LBS were grown in MRS broth (De Man, Rogosa, Sharpe; Guangdong Huangkai Science & Technology Co., Ltd., Guangzhou, China) under anaerobic condition at 37°C for 24 h without shaking.

*Escherichia coli* K1, a rifampicin-resistant *E. coli* RS218 (O18:K1:H7), was kindly provided by Prof. Sheng-He Huang (University of Southern California, United States). *E. coli* K1 was isolated from the cerebrospinal fluid of a neonate with meningitis (Weiser and Gotschlich, 1991). The bacterial pathogen, *E. coli* K1 was incubated at 37°C with constant shaking at 200 revolutions per minute (rpm) in brain-heart infusion (BHI) broth (Guangdong Huangkai Science & Technology Co., Ltd., Guangzhou, China) with rifampicin (100 μg/ml) for 14 h.

The human colon carcinoma cell lines HT-29 was purchased from Shanghai Institute of Cell Biology (Shanghai, China) and routinely cultured in RPMI 1640 medium (Gibco, Carlsbad, CA, United States) with 10% heat-inactivated fetal bovine serum (PAN, Adenbach, Bavaria), 1% antibiotic mixture (100 mg/ml streptomycin and 100 U/ml penicillin G; Hyclone, Logan, Utah) at 37°C in a humidified 5% CO_2_ atmosphere.

### Adhesion and Invasion Assays

HT-29 was cultured in 24-well plates (Corning, United States) and pre-incubated/co-incubated with LBS and/or *E. coli* K1 for 3 h. After incubation, 10^7^ colony forming units (CFU) of *E. coli* K1 were added to the HT-29 monolayers and further incubated for 2 h at 37°C. The adhesion and invasion assays were performed as follows, respectively. After *E. coli* K1 infection, monolayers were washed three times with RPMI 1640 medium and lysed by 0.5% Triton X-100 for 8 min (Yu et al., 2015). Number of cell-associated bacteria (recovered from the lysing cells) was counted after 16 h of incubation in the BHI agar plates with rifampicin (100 μg/ml). Each assay was carried out in triplicate wells and repeated at least three times.

Invasion assays were carried out after incubation with bacteria. HT-29 monolayers were washed three times and incubated with a medium containing gentamicin (100 μg/ml) for 1 h at 37°C to eliminate extracellular bacteria (Yu et al., 2015). Then cells were washed and the number of internalized bacteria recovered from the lysing cells was enumerated as described above. Each assay was carried out in triplicate wells and repeated at least three times.

### Protein Extraction and Western Blotting Analysis

HT-29 cells were cultured in 6-well plates and grown to complete confluence. The HT-29 monolayers were allocated into four groups: I (PBS), II (LBS), III (*E. coli* K1), and IV (LBS plus *E. coli* K1). Group I and III were pre-treated with phosphate buffer saline (PBS) while II and IV were pre-treated with LBS (1 × 10^8^ CFU) for 3 h at 37°C. Then, III and IV were incubated with *E. coli* K1 (1 × 10^8^ CFU) while I and II were given PBS for 3 h. After this, cells were washed with cold PBS for three times and lysed with 150 μl radioimmunoprecipitation assay (RIPA) buffer (Beyotime Institute of Biotechnology, Shanghai, China) containing 1% Cocktail and 1 mmol/L phenylmethanesulfonyl fluoride. The lysing samples were centrifuged at 10000 rpm for 5 min at 4°C and supernatant was collected. The concentration of protein was determined by the bicinchoninic acid (BCA) assay (Solarbio Science & Technology Co., Ltd., Beijing, China). All samples were diluted with 5 × SDS loading buffer and denatured at 100°C for 10 min. Equal amounts of proteins (20 μg) were electrophoresed on SDS-polyacrylamide gels and electro-transferred to polyvinylidene difluoride membrane (Millipore, United States). The membranes were blocked for 1 h at room temperature with 5% skim milk (dissolve in PBS + 0.05% Tween 20, PBST) and incubated with primary antibody occludin, MUC2 (Abcam, Cambridge, United Kingdom), ZO-1 (Proteintech, Wuhan, China) or β-actin (Bioss, Beijing, China). After this, membranes were washed three times and incubated with horseradish peroxidase (HRP)-coupled secondary antibody (Dingguo, Beijing, China). The results were observed and analyzed using an enhanced chemiluminescence reagent kit (Bio-Rad Laboratories, United States) and Tanon-6200 gel imaging and analysis software. Each assay was repeated at least three times.

### Periodic Acid-Schiff (PAS) Assay

We analyzed mucin content using PAS assay as described as previously (Garcia et al., 2009). HT-29 cells were cultured in 6-well plates and treated as mentioned in the western blotting assay. Then treated cells were collected and lysed using RIPA buffer to obtain soluble ingredients. Each sample was placed in the 96-well plate (10 μl/well) followed by added 200 μl 0.1% periodic acid (Solarbio Science & Technology Co., Ltd., Beijing, China). The plate was incubated for 2 h at 37°C, then 200 μl of Schiff’s reagent (Solarbio Science & Technology Co., Ltd., Beijing, China) was added and incubated at room temperature for half an hour. Optical density (OD) of the resulting solution was detected at 555 nm wavelengths and taken as a measure of the amount of PAS positive product. Each assay was performed in triplicate wells and repeated three times.

To explore the morphological alterations of mucin layers during LBS and/or *E. coli* K1 treatment, HT-29 monolayers were cultured in 24-well plate and treated as described in the western blotting assay. Then monolayers were fixed with 4% paraformaldehyde at 4°C overnight and stained using a PAS kit (Solarbio Science & Technology Co., Ltd., Beijing, China) according to the manufacturer’s instruction. The results were observed and analyzed using light microscopy.

### Immunofluorescence of MUC2

HT-29 monolayers were cultured in 24-well plates and treated as described in the western blotting assay. Monolayers were washed with PBS and fixed in 4% paraformaldehyde at room temperature for 10 min. Then cells were blocked with 1% bovine serum albumin (BSA) in PBST for 30 min followed by incubation with rabbit anti-MUC2 antibody overnight at 4°C. Cells were washed four times with PBST and incubated with DyLight 488-conjugated goat anti-rabbit IgG antibody (Abbkine, United States) at room temperature for 1 h followed by incubation with Hoechst 33342 for 10 min. Results were observed using fluorescence microscopy (Nikon Eclipse: TE 2000-E, Japan). Each assay was performed in triplicate wells and repeated three times.

### Bacterial Translocation and Transepithelial Permeability Measurements

HT-29 cells were cultured in the Transwell insert (6.5 mm diameter, 3 μm pore size, Corning Costar Corp., United States) and divided into four groups: I (PBS), II (LBS), III (*E. coli* K1), and IV (LBS plus *E. coli* K1). Group I and III were pre-treated with PBS while II and IV were pre-treated with LBS (2 × 10^5^ CFU) for 3 h at 37°C. Three hours later, PBS and *E. coli* K1 (2 × 10^5^ CFU) were added to the upper chamber of Transwell inserts in I and II, III and IV group, respectively, and incubated for 2 h at 37°C. After this, HRP was added to the upper chamber of all the Transwell inserts and incubated for 1 h at 37°C. Afterward, 20 μl medium were extracted from the lower chamber, one half was transferred into 96-well plate for testing the OD_450_ value of HRP, and the another half was diluted and plated on BHI agar plates for bacterial number counting. Each assay was performed in triplicate wells and repeated three times.

### Cell Cytotoxicity Assay

Cells were cultured in 96-well plate and treated as described in the western blotting assay. HT-29 cell damage was detected using Lactate Dehydrogenase (LDH) activity assay according to the manufacturer’s instruction of LDH detection kit (Suzhou Keming Science & Technology Co., Ltd., Suzhou, China) (Borosmajewska et al., 2015). Briefly, LDH activity was tested in both the culture supernatant and adherent cells. LDH content from the culture supernatant was defined as index of cytotoxic cells, while LDH from the adherent cells represented the total LDH. LDH content was tested by microtitre plate reader (wavelength: 572 nm), the percent cytotoxicity was calculated as follows: %Cytotoxicity = [(Experimental sample-Background)/Total LDH release] × 100. Total LDH release was achieved by lysing cells in 1% Tritonx-100. The data were expressed as a percentage of the control (untreated HT-29 cells).

### Cell Apoptosis Assay

Cellular apoptosis was tested by Hoechst 33342/propidium iodide (PI) double staining method (Nie et al., 2016). Cells were cultured in 24-well plates and treated as described in western blotting assay. In brief, 1 μg/ml PI (Biosharp, Hefei, China) and 2 μg/ml Hoechst 33342 (Solarbio Science & Technology Co., Ltd., Beijing, China) were added into each well and co-incubated for 20 min in the dark room. To minimize background, cells were washed with PBS for three times after incubation. Results were observed using a fluorescence microscopy (Nikon Eclipse: TE 2000-E, Japan). Each assay was performed in triplicate wells and repeated three times.

### Neonatal Rat Model of Gut-Derived *E. coli* K1 Infection

We used the neonatal rat model to explore how LBS exert the prophylactic effect against neonatal *E. coli* K1 translocation similar with our recent research (Yu et al., 2015). Twenty pathogen-free Sprague-Dawley rat pups (2 days old) were divided randomly into four groups: I (PBS), II (LBS), III (*E. coli* K1), and IV (LBS plus *E. coli* K1). Pups were given LBS (1 × 10^9^ CFU) or PBS by oral gavage once a day for 3 days. Afterward, all pups in groups III and IV received 5 × 10^9^ CFU of *E. coli* K1 with the same delivery approach. Two days after *E. coli* K1 infection, all pups were anaesthetized with ketamine and lidocaine, the intestinal tissues were removed and fixed in 4% paraformaldehyde, and embedded in paraffin for immunohistochemical analysis. Intestinal colonization and bacteremia were determined as described previously via culturing the stool and blood samples, respectively, on Luria-Bertani agar plates with rifampin (50 μg/ml) (Yu et al., 2015).

### PAS Staining and Immunohistochemical Staining

Paraffin-embedded tissue sections were stained using PAS stain kit according to manufacturer’s instruction. The PAS-stained sections were counterstained with hematoxylin and observed using light microscopy.

For immunohistochemical staining, paraffin-embedded tissue sections were deparaffinized and the antigen was retrieved. Sections were blocked with normal goat serum and incubated at 4°C with primary antibody specific for Ki67, IgA, and ZO-1, respectively. Then the sections were incubated with HRP-conjugated second antibodies with 50 mM Tris-HCl buffer (pH = 7.4) containing DAB (3,3 = -diaminobenzidine) and counterstained with hematoxylin. Images were obtained using a light microscopy. Digital images were collected from at least three different fields per section and quantified by NIH image analysis software (Image J).

### Intestinal Permeability Assay

Intestinal permeability was assessed by fluorescein isothiocyanate (FITC)-dextran leakage assay. Neonatal rats were treated as described above. Forty-four hours after *E. coli* K1 infection, all pups were gavaged with FITC-dextran (600 mg/kg, MW 4000, Santa Cruz, CA, United States). Four hours later, all pups were anaesthetized with ketamine and lidocaine. Blood was collected through heart puncture and FITC concentrations were measured using a fluorescence spectrophotometer.

### Statistical Analysis

All experiments were performed at least three times and the data were expressed as mean ± standard deviation (SD). Unpaired *t*-test was used to analyze the difference between two groups, one-way analysis of variance (ANOVA) was used for analyzing the difference among three or more groups. All analyses were performed by Graphpad Prism 5 software. *P* < 0.05 was considered to be statistically significant.

## Results

### Pre-treatment with LBS Mitigates *E. coli* K1-Induced Down-Regulation of Mucin

**FIGURE 1:**
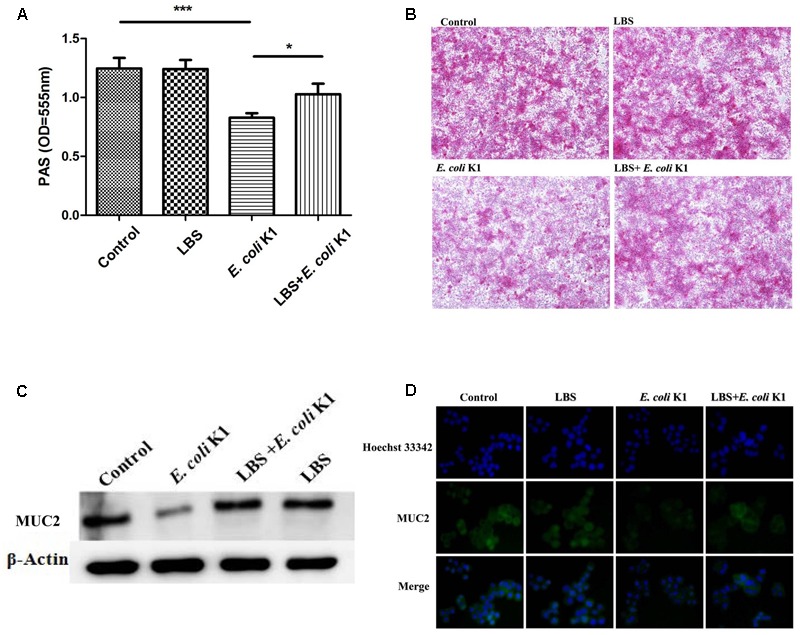
*Lactobacillus bulgaricus, Bifidobacterium*, and *Streptococcus thermophilus* (LBS) alleviates mucin degradation during *Escherichia coli* K1 infection. HT-29 cells were pre-treated with or without LBS before *E. coli* K1 infection. Cells treated with PBS or LBS alone were served as controls. Cellular proteins were extracted for **(A)** periodic acid-Schiff (PAS) assay and **(C)** western blot analysis. **(B)** HT-29 monolayers were stained with PAS and observed under light microscope (100× magnification). **(D)** Immunofluorescence of MUC2 was obtained by fluorescence microscopy (200× magnification), green staining represents the MUC2, the cell nuclei were stained with Hoechst 33342 (blue). Results are represented as mean ± SD. ^∗^*P* < 0.05, ^∗∗∗^*P* < 0.001.

Golden Bifid is a well-known trade name of a complex-probiotic-preparation, which containing living *Bifidobacterium longum, L. bulgaricus*, and *S. thermophilus*. As probiotic property is characteristic of some specific strain, we have performed real time PCR to assure that, these strains are existing in MRS broth. Our results suggested that all these three strains were included under condition of growth in MRS broth (data not shown). Previous study reported that *E. coli* K1 was able to degrade mucin layer on the intestinal surface (Valeri et al., 2015). As the mucin layer is the first barrier that *E. coli* K1 encounter in the intestine (Corfield et al., 2001; Kim and Ho, 2010), we initially explored the possibility of LBS protecting the mucin from *E. coli* K1-induced degradation. As shown in **Figure 1A**, infection with *E. coli* K1 significantly down-regulated the production of mucin. In contrast, pre-treatment with LBS could alleviate *E. coli* K1-induced loss of mucin. Similar results were observed with morphological alterations of mucin layers. As shown in **Figure 1B**, PBS or LBS treated HT-29 monolayers were covered with a purple and thick mucin layer. However, infection with *E. coli* K1 led to a lilac, thin and fractured mucin. In contrast, incubation with LBS before *E. coli* K1 infection could prevent the *E. coli* K1-induced disruption of the mucin layer. Mucin2 (MUC2) is the major components of the intestinal mucosa, we further performed immunoblotting and immunostaining to examine the expression of MUC2 in HT-29 cells during stimulation with LBS and/or *E. coli* K1. As expected, infection with *E. coli* K1 could decrease the MUC2 expression while pre-treatment with LBS was able to reverse *E. coli* K1-mediated mucin depletion (**Figures 1C,D**).

### LBS Inhibits Adhesion/Invasion of *E. coli* K1 to HT-29 Cells

**FIGURE 2:**
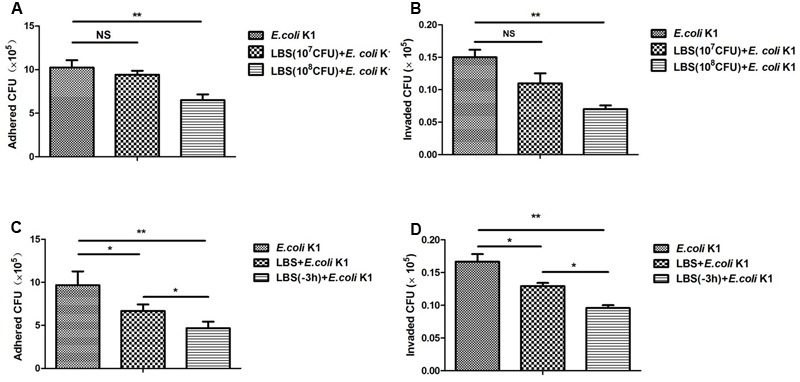
*Lactobacillus bulgaricus, Bifidobacterium*, and *Streptococcus thermophilus* inhibits adhesion and invasion of *E. coli* K1 to HT-29 cells. HT-29 cells were cultured in 24-well plate and pre-treated with various doses of LBS for 3 h before adding *E. coli* K1 or co-incubated with LBS plus *E. coli* K1. Adhesion and invasion assays were carried out as described in section “Materials and Methods.” The numbers of associated bacteria **(A,C)** and intracellular bacteria **(B,D)** were determined. Results are represented as mean ± SD. ^∗^*P* < 0.05, ^∗∗^*P* < 0.01.

Adhesion and invasion to intestinal epithelium are the pivotal steps in intestinal bacterial translocation and thereafter to enter the circulation resulting in a systemic infection. We next determined whether LBS has the inhibitory effect on adhesion and invasion of *E. coli* K1. Two sets of experiments were performed. In one set, HT-29 cells were stimulated simultaneously with LBS (10^7^ or 10^8^ CFU/well) and *E. coli* K1 (10^7^ CFU/well) (**Figures 2A,B**). In another set, HT-29 cells were pre-incubated with LBS for 3 h (-3 h indicated in **Figures 2C,D**) before exposure to *E. coli* K1 or co-incubation with LBS and *E. coli* K1, this set was established to explore whether the inhibitory effect of pre-treatment with LBS was superior to co-treatment. As shown in **Figures 2A–D**, in both the pre- and co-incubation group, lower number of *E. coli* K1 CFU were observed in LBS-treated groups as compared with that of control. Interestingly, we found the number of *E. coli* K1 CFU recovered from pre-incubated group was fewer compared with that of co-incubated group (**Figures 2C,D**). Together, these results suggested that LBS could inhibit adhesion and invasion of *E. coli* K1 to intestinal cells, and the preventive effect of LBS is superior to its therapeutic effect.

### LBS Could Attenuate the *E. coli* K1-Induced Cytotoxicity and Apoptosis in HT-29 Cells

**FIGURE 3:**
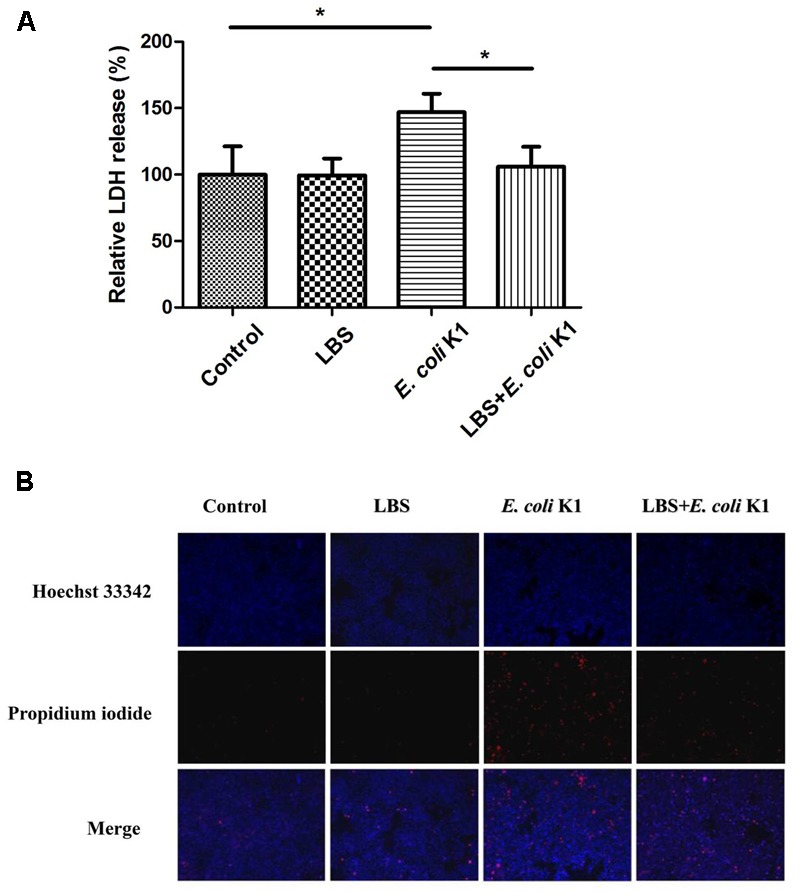
Pre-treatment with LBS decreases *E. coli* K1-induced damage and apoptosis of HT-29 cells. HT-29 cells were treated as described in **Figure 1**. **(A)** LDH activity was analyzed by microtiter plate reader and the results were expressed as a percentage of the control (untreated cells). Results are presented as mean ± SD. ^∗^*P* < 0.05. **(B)** HT-29 cells were illustrated by fluorescence microcopy (100× magnification). Hoechst 33342 was used to stain nuclei of all cells (blue), PI was used to detect the dead cells (red).

Induction of intestinal epithelial cells damage and apoptosis is an important approach for *E. coli* K1 translocation across the gut barrier (Pietzak et al., 2004). It is interestingly to determine whether the pre-treatment with LBS could alleviate *E. coli* K1-induced damage and apoptosis of enterocyte. LDH is an intracellular enzyme that is released from the cells after it is subjected to membrane damage (Soman et al., 2008). We used the LDH activity assay to explore the protective effect of LBS on HT-29 cells. As shown in **Figure 3A**, no significant difference in LDH activity was observed after LBS treatment, while cells exposed to *E. coli* K1 showed a significant increase in LDH activity. In contrast, pre-treatment with LBS could decrease the LDH release caused by *E. coli* K1 infection.

Hoechst 33342 and PI double staining are routinely used to distinguish live and dead cells. When cells are in the late apoptotic stage or in the early necrotic stage, the nuclei are red in color, whereas the nuclei of live cells appear blue. In **Figure 3B**, Hoechst-PI staining showed that *E. coli* K1 could induce the apoptosis of cells and pretreatment with LBS reversed it. These results suggested that pre-treatment with LBS could alleviate *E. coli* K1-induced cell death and apoptosis.

### Pretreatment with LBS Inhibits *E. coli* K1-Induced Disruption of Intestinal Integrity and Translocation

**FIGURE 4:**
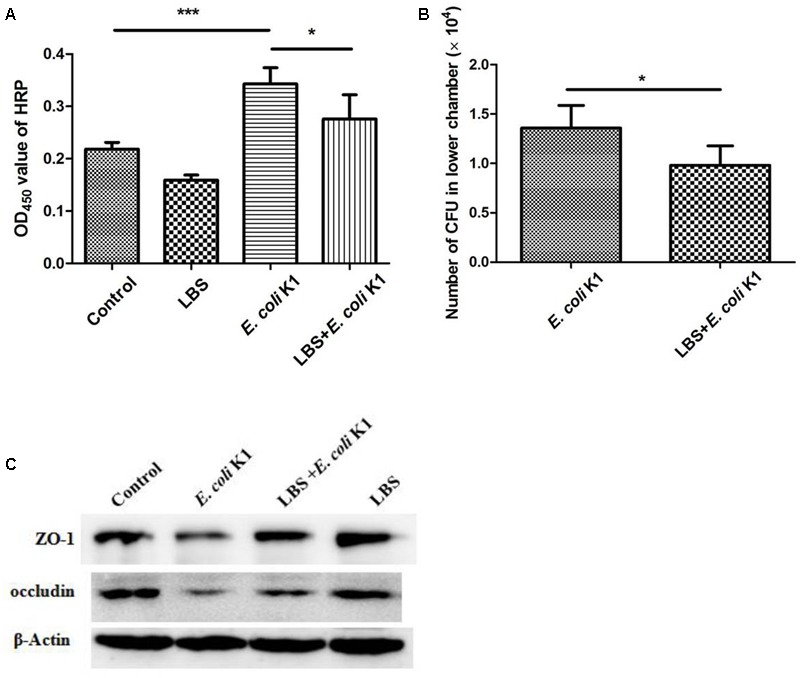
*Lactobacillus bulgaricus, Bifidobacterium*, and *Streptococcus thermophilus* prevents *E. coli* K1-induced disruption of intestinal integrity. HT-29 cells were cultured on the upper chamber of the Transwell insert and pre-treated with or without LBS before *E. coli* K1 infection. Cells treated with PBS or LBS alone were served as controls. After infection, HRP was added to the upper chamber of Transwell for 1 h. Bacteria and HRP translocated from the upper chamber to the lower chamber were quantified as described in section “Materials and Methods.” **(A)** The OD_450_ value of HRP. **(B)** The number of *E. coli* K1 CFU, which penetrated across the HT-29 monolayer. Results are represented as mean ± SD. ^∗^*P* < 0.05, ^∗∗∗^*P* < 0.001. **(C)** HT-29 cells were treated as described in **Figure 1**, proteins of each group were isolated for western blotting. The expressions of ZO-1 and occludin was determined, β-Actin band was used as an indicator of protein loading.

Tight junctions seal the intercellular space of intestinal epithelial cells to form a contiguous and relatively impermeable barrier that regulates the passage of antigen through the paracellular pathway. However, pathogen such as *E. coli* K1 could compromise intestinal integrity and translocation across the intestinal barrier into the blood stream, leading to systemic infection (Valeri et al., 2015). We next tested whether LBS could modulate the intestinal integrity during *E. coli* K1 infection. As shown in **Figure 4A**, in contrast to the untreated HT-29 monolayer (control), cells infected with *E. coli* K1 alone developed higher concentration of HRP, indicating that *E. coli* K1 could impair the intestinal integrity, and consequently led to intestinal barrier leakage. Interestingly, this obvious increase of HRP leakage could be significantly prevented by pre-treatment with LBS. Meanwhile, the number of bacteria translocated from the upper chamber to the lower chamber, which was higher in only *E. coli* K1 infection group, also decreased in LBS pre-treated group (**Figure 4B**). These findings suggested that LBS was able to inhibit *E. coli* K1-induced disruption of intestinal integrity and block its translocation.

In order to further explore the protective effect of LBS on intestinal integrity, we performed immunoblotting assay to detect the expressions of two main tight junction proteins, ZO-1 and occludin. As expected, pretreatment with LBS could attenuate ZO-1 and occludin degradation during *E. coli* K1 infection (**Figure 4C**). These results indicated that LBS could reduce *E. coli* K1-mediated injury of intestinal integrity and thereby prevent from intestinal translocation of *E. coli* K1.

### Administration with LBS Prevents *E. coli* K1-Induced Dysfunction of Intestinal Defense in Neonatal Rats

**FIGURE 5:**
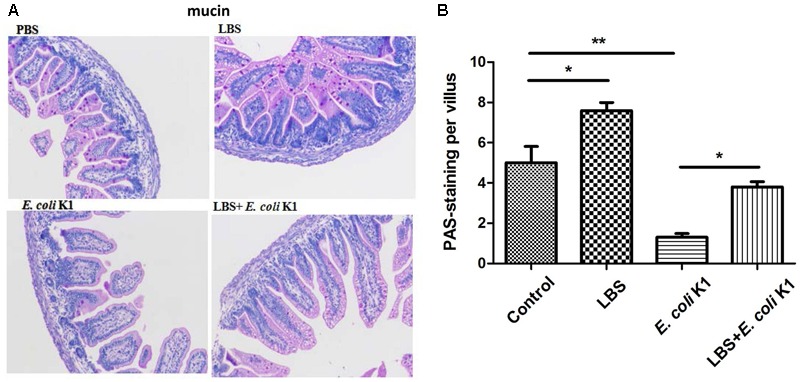
Effects of LBS on mucin expression of neonatal colon tissue with or without *E. coli* K1 infection. Two days old neonatal rats were orally administered with or without LBS for 3 days before *E. coli* K1 infection. Pups administered with PBS or LBS alone was served as controls. **(A)** Colon tissues received different treatment were processed with PAS stain and visualized by light microscopy (200× magnification). **(B)** The number of PAS-positive cells was calculated. Results are represented as mean ± SD. ^∗^*P* < 0.05, ^∗∗^*P* < 0.01.

**FIGURE 6:**
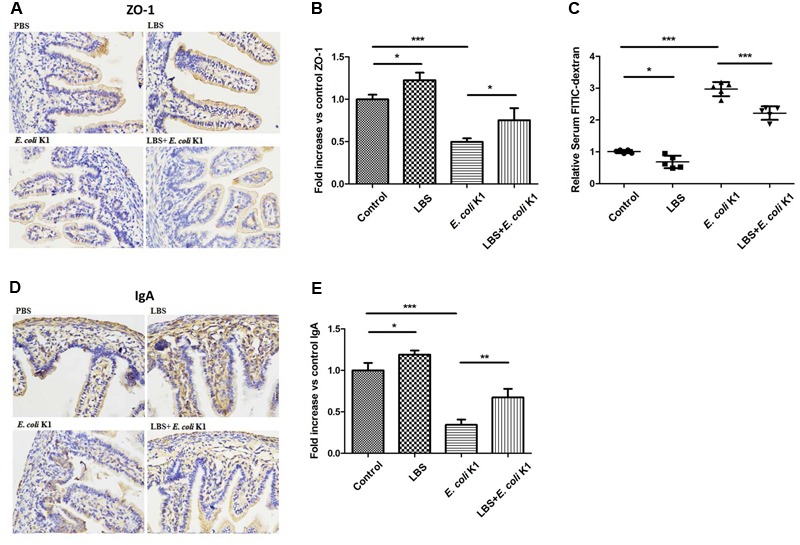
Effects of LBS on ZO-1, IgA expressions and the permeability of neonatal intestinal tract with or without *E. coli* K1 infection. Two days old neonatal rats were treated as described in **Figure 5**. Paraffin-embedded small intestinal tissues were prepared for ZO-1 **(A)** and IgA **(D)** staining. Images were visualized by light microscopy (400× magnification). Semi-quantitative analysis of ZO-1 **(B)** and IgA **(E)** were performed by Image J. **(C)** The intestinal permeability was measured by FITIC-dextran leakage assay. The value of PBS group was assigned as 1. Data were represented as mean ± SD, *n* = 5. ^∗^*P* < 0.05, ^∗∗^*P* < 0.01, ^∗∗∗^*P* < 0.001.

Next, we used the animal models of neonatal rats *E. coli* K1 bacteremia and meningitis to explore how LBS exerts prophylactic effect against neonatal gut-derived *E. coli* K1 infection. In this animal model, the course of *E. coli* K1 infection mimics the natural route of intestinal colonization, translocation, bacteremia, sepsis and meningitis found in the human neonate. As shown in **Table 1**, the colonization of *E. coli* K1 in the intestinal tract and bacteremia incidence of LBS-treated pups was decreased significantly compared with that of non-treated pups. These results indicated again that LBS could prevent neonatal *E. coli* K1 translocation. As mucin, IgA, ZO-1 and intestinal barrier functions are important for intestinal defense against bacterial translocation (Jakaitis and Denning, 2014), we performed immunohistochemical staining and FITC-dextran leakage assay to determine whether stimulation with *E. coli* K1 could impair these intestinal defenses. As shown in **Figure 5**, PAS staining in intestinal tissue slice revealed that, infection with *E. coli* K1 could decrease the PAS-positive cells per villus compared with the control (PBS-treated pups). Similarly, **Figure 6** showed that *E. coli* K1 infected pups revealed a lower expression level of ZO-1 and IgA as well as a higher intestinal permeability. However, when pre-treated with LBS, all these detrimental effects of *E. coli* K1 were reversed (**Figures 5, 6**). These data suggested that alleviating the detrimental effects of *E. coli* K1 on intestinal defenses could explain the preventive role of LBS against neonatal *E. coli* K1 translocation.

**Table 1:** The population of *Escherichia coli* K1 colonization and incidence of bacteremia.

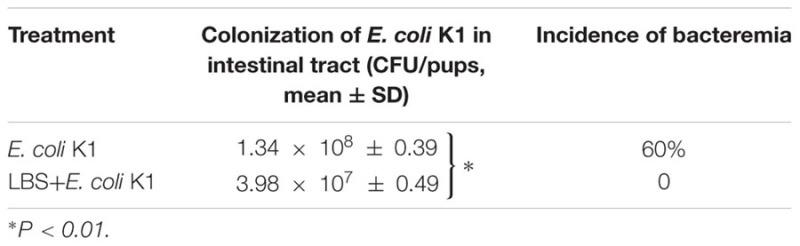

As mentioned in the Introduction section, intestinal barrier function always not fully developed in the early neonatal period, which is the important reason responsible for neonatal susceptibility to *E. coli* K1 translocation. A previously published study reported that, the commensal and probiotic bacteria might prevent neonatal necrotizing enterocolitis by maturing intestinal defenses (Jakaitis and Denning, 2014). Is it interestingly to explore whether LBS could promote the formation of neonatal intestinal defense. To explore this issue, we compared the expression levels of mucin, IgA and ZO-1, and intestinal permeability between LBS-treated and non-treated pups. Results showed that treatment with LBS could not only increase mucin, IgA and ZO-1 expression, but also decrease intestinal permeability (**Figures 5, 6**). These results indicated that LBS could promote maturation of neonatal intestinal defenses.

### LBS Pre-treatment Restores the Proliferative Capacity of Epithelial Cells in *E. coli* K1 Infected Small Intestine

**FIGURE 7:**
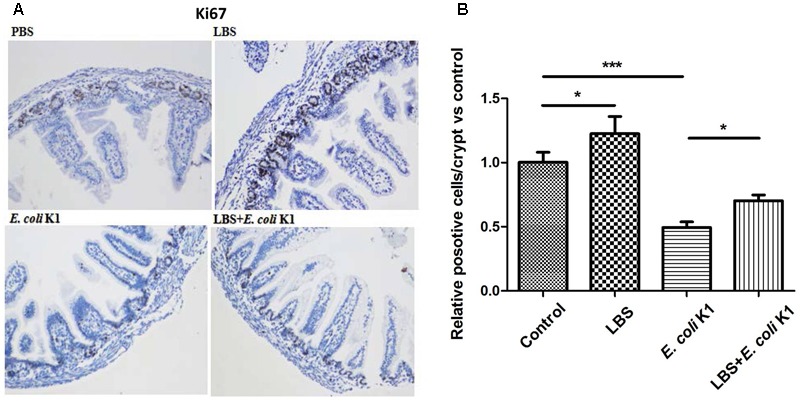
Effects of LBS on proliferation of neonatal intestinal epithelium with or without *E. coli* K1 infection. Two days old neonatal rats were treated as described in **Figure 5**. **(A)** Small intestines were processed for Ki67 immunohistochemistry. Images were visualized by light microscopy (200× magnification). **(B)** Ki67-positive cells were calculated. The value of PBS group was assigned as 1. Results are represented as mean ± SD. ^∗^*P* < 0.05, ^∗∗∗^*P* < 0.001.

We further investigated whether *E. coli* K1 infection could affect the capacity of ileal cells to repair the damaged intestinal epithelia. Ki67 staining was used to detect mitotic cells. We found fewer numbers of Ki67-positive dividing cells in *E. coli* K1 infected pups compared with the PBS and LBS treated pups (**Figures 7A,B**). In contrast, pretreatment of rats with LBS before *E. coli* K1 infection could restore the number of Ki67-positive dividing cells. Interestingly, we found that LBS treated pups also had more Ki67-positive dividing cells than that of non-treated pups, indicated that LBS was able to promote intestinal epithelial cell proliferation.

## Discussion

**FIGURE 8:**
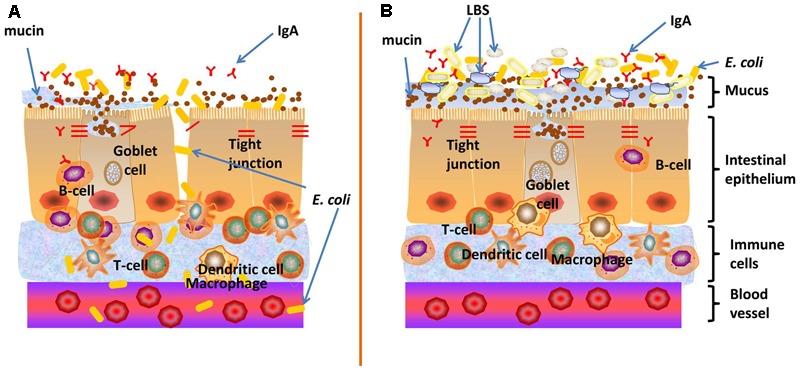
Proposed mechanisms on how LBS exert preventive effect against neonatal *E. coli* K1 translocation. **(A)** The intestinal barrier function of neonates is not fully developed, which may provide opportunities for *E. coli* K1 translocation across the gut barrier into the blood stream, leading to bacteremia and meningitis. **(B)** LBS promote the formation of neonatal intestinal barrier function, including enhancement of mucin and IgA productions and up-regulation of the expression of ZO-1 to seal the intercellular space of intestinal epithelial cells (reflected by a decrease in the intestinal permeability). This multifactorial approach enhances intestinal barrier functions and thereby limits bacterial translocation across the neonatal intestinal mucosa.

More than a simple anatomical structure, the gut barrier represents a functional unit, which plays a key role in maintenance of intestinal homeostasis and prevention of gastrointestinal and extra-intestinal diseases. *E. coli* principally resides in the gut, but could also translocate to distant organs such as the bladder and brain, where they can cause urinary tract infections and meningitis. We have recently showed that LBS-inhibited *E. coli* K1 translocation across the intestinal barrier was mediated by MUC2 (Yu et al., 2015). In this study, we further discovered that exposure to *E. coli* K1 could impair gut barrier function as reflected by inducing intestinal epithelial apoptosis and injury to various intestinal defenses including mucin, IgA and tight junction proteins expression as well as integrity of the gut barrier. However, pretreatment with LBS could protect gut barrier from intrusion of *E. coli* K1. Furthermore, we found that LBS-treated neonatal pups have a more mature intestinal defense, as reflected by higher expression of mucin, ZO-1, IgA and lower intestinal permeability than that of non-treated pups. These data suggested that neonatal treatment with LBS promotes the formation of the intestinal epithelial barrier and confer a high resistance to *E. coli* K1 insult, and thereby prevent its translocation (**Figure 8**).

The intestinal physical barrier consists of the gut microbiota, mucous layer, intestinal epithelial layer and the epithelial junction adhesion complex. A single layer of intestinal epithelium is composed of enterocytes, goblet cells, paneth cells and microfold cells (Watson et al., 2014). These cells contribute to the formation of intestinal barrier according to its specific differentiation characteristics, including secretion of the mucin, production of antimicrobial peptides, formation of tight junction and antigen presentation and so on. Thus, intestinal epithelium is a key component in the arsenal of defense mechanisms required to prevent bacterial translocation. Human colon carcinoma cell lines HT-29 is a valuable tool for studies of intestinal epithelial function *in vitro*, as it could express differentiation features, including production of mucus and formation of steady intercellular tight junction (Rousset, 1986). Using this model, we established that LBS has multiple protective role on epithelial barrier function during *E. coli* K1 infection, including: (1) blockage of *E. coli* K1-caused loss of mucin layer; (2) inhibition of adhesion and invasion of *E. coli* K1 to intestinal cells; (3) alleviation of *E. coli* K1-induced epithelial cell damage and apoptosis; (4) modulation of integrity of intestinal barrier. To the best of our knowledge, this is the first study reported regarding the multiple protective effects of probiotics on intestinal physical barrier during *E. coli* infection. It is possible that a mixture of probiotics may beneficially impact the overall profile of intestinal barriers, and different probiotic strains may confer synergistic benefits to intestinal epithelial functions. This hypothesis is supported by many previous experimental studies, which indicated that a single probiotic strain has failed to exert its preventive role on bacterial translocation (Bauer et al., 2002; Soriano et al., 2012). Furthermore, it would be interesting and informative to explore whether the dose of multi-strain probiotic is lower than that of single strains for preventing neonatal bacterial translocation. Although, normally non-pathogenic, probiotics could also exhibit potential harmful effects in high doses in neonates and preterm babies (Cilieborg et al., 2011; Li et al., 2012; Patel et al., 2012).

The barrier function of the intestinal mucosa is immature in newborns, especially in low birth weight infants and preterm babies. Undeveloped intestinal defenses in the neonatal period may provide opportunities for pathogenic translocation across the gut barrier into the blood stream, leading to systemic inflammation and infection. It has been established that the mature intestine has many physical barriers to prevent the establishment of pathogenic bacteria, such as peristalsis, microbial antagonism by commensal microflora, cell surface mucus and IgA, and tight junctions between intestinal epithelial cells. All these are designed to limit bacteria to the gut lumen and prevent attachment and translocation across the intestinal epithelium. However, the mature intestinal defense, which is equipped with dense intestinal mucin and IgA layer, stable tight junction and low intestinal permeability, always not fully developed in early neonatal period (Claud, 2009; Patel et al., 2012; Birchenough et al., 2013). In the present study, we found that LBS could increase the expression of mucin, IgA and ZO-1 in the intestinal tissue of neonatal rats as well as decreased the intestinal permeability. Furthermore, LBS could also promote proliferation of neonatal enterocytes. These data suggested that intensifying the development of neonatal intestinal defense could be the underlying mechanism to explain the contribution of LBS to neonatal resistance against gut-derived *E. coli* K1 infection.

Gut microbiome is largely subjected to dynamic changes after birth and plays an important role within our body, such as modulation of many metabolic pathways, nutrient and drug metabolism, maintenance of epithelial integrity, regulation of gastrointestinal peristalsis and maturation of the intestinal immune system (Sommer and Bäckhed, 2013; Deshmukh et al., 2014). The alteration of gut microbiota is often associated with several disorders, such as inflammatory bowel disease, metabolic syndrome, obesity and irritable bowel syndrome (Kronman et al., 2012; Cammarota et al., 2014). A recent study has demonstrated that a balanced intestinal microbiota plays a key role in neonatal resistance to *E. coli* K1 sepsis (Deshmukh et al., 2014). Thus, the limit of the present study is not to explore the effect of LBS on the colonization and formation of neonatal intestinal commensal bacteria, which have been proved to of a very important role on the fight with *E. coli* K1 infection (Deshmukh et al., 2014). We speculated that LBS could promote development of neonatal intestinal microbiota. This hypothesis is based on two rational bases. Firstly, previous study showed that mucus could provide glycan-dependent anchoring sites and nutrients to intestinal commensal microbiota besides protecting the enterocyte from pathogenic assault (Yan et al., 2016). Thus, LBS may help in the colonization of commensal bacteria by inducing mucin production. Secondly, IgA is a dominant Ig isotype that plays a critical role in intestinal immune function (Wittig and Zeitz, 2003). It has been established that IgA promotes the establishment of microbiota composition and maintains the diversity of microbiota (Fransen et al., 2015). Moreover, research has shown that IgA is able to exclude the pathogenic microbes and control the commensal microbes by maintaining the homeostasis of gut flora (Macpherson et al., 2008). Thus, inducing IgA expression by LBS may play an important role in formation, development and maintenance of intestinal microbiota composition. Another important issue is whether the culture supernatant of LBS exerts protective role on neonatal intestinal mucous. More recently, probiotic-derived soluble factors (defined as “postbiotics”) have been suggested to have beneficial properties as same as their original “parent”-live probiotics (Tsilingiri et al., 2012). Postbiotics have been implicated to exhibit a beneficial effect on intestinal barrier through inducing mucin production, maintenance of intestinal barrier integrity and in promoting immune function (Tsilingiri and Rescigno, 2012). Furthermore, it has been established that in normal flora, *Bifidobacterium* and *Lactobacillus* could directly exert antibacterial effects on pathogens by producing bacteriocins and toxic acidic substances (Servin, 2004). Thus, it is reasonable to speculate that LBS culture supernatant may also exhibit the preventive effects toward infectious pathogens. Actually, during the initial experiment, we have assessed the antimicrobial activity of LBS *in vitro* by growing *E. coli* K1 in the presence of LBS. *E. coli* K1 were incubated with 1 × 10^9^ CFU of LBS in BHI broth or cell culture media (RPMI 1640 + 1% fetal bovine serum) for 1 to 8 h at 37°C. The bacterial growth curves were determined by measuring the OD_600_ at each time point. The results showed that LBS is unable to inhibit *E. coli* K1 multiplication when co-incubation time is less than 4 h. In contrast, the killing effect was revealed if the co-incubation time is more than 4 h. These results indicated that, in our study, the LBS-mediated inhibitory effect on *E. coli* K1 infection *in vitro* may not be via its antibacterial agent. It is possible that the incubation time less than 4 h is not enough for LBS mixture to secrete antibacterial agent, such as bacteriocins. However, we could not rule out the possibility that LBS-secreted antibacterial agents have no positive effect *in vivo*. Furthermore, to the best of our knowledge, there is no study aimed to investigate the health effects of postbiotics come from probiotics mixture. Thus, our ongoing work will involve the beneficial effect of LBS culture supernatant.

In summary, our study suggested that LBS enhanced neonatal resistance to *E. coli* K1 infection could be mediated through maturation of neonatal intestinal defense. Our data also indicated that LBS have great potential to act as an effective prophylaxis for immature intestine-associated diseases, such as inflammatory bowel disease, infectious enteritis and necrotizing enterocolitis. Hopefully, understanding the effect and mechanism of probiotics on basic intestinal pathophysiology will influence how they will be used clinically in the future.

## Author Contributions

HC, QZ, XH, SP, and S-HH conceived and designed the experiment. QZ, XH, HX, ZG, LC, and HT performed the experiment. XH, QZ, SB, SP, HX, ZG, HT, LC, and SH analyzed the data. SH contributed reagents/materials/analysis tools. QZ, XH, SP, SB, SH, and HC participated in its design and coordination and helped to draft the manuscript. All authors read and approved the final manuscript.

## Conflict of Interest Statement

The authors declare that the research was conducted in the absence of any commercial or financial relationships that could be construed as a potential conflict of interest.

## References

[B1] Bauer T. M. Fernández J. Navasa M. Vila J. Rodés J. (2002). Failure of *Lactobacillus* spp. to prevent bacterial translocation in a rat model of experimental cirrhosis. *J. Hepatol.* 36 501–506. 10.1016/S0168-8278(02)00003-X 11943421

[B2] Bhagat R. Hussain S. Q. Gattoo I. A. Wani S. A. (2015). Incidence of meningitis in late onset sepsis. *Int. J. Contemp. Pediatr.* 2 96–102. 10.5455/2349-3291.ijcp20150507

[B3] Birchenough G. M. Johansson M. E. Stabler R. A. Dalgakiran F. Hansson G. C. Wren B. W. (2013). Altered innate defenses in the neonatal gastrointestinal tract in response to colonization by neuropathogenic *Escherichia coli*. *Infect. Immun.* 81 3264–3275. 10.1128/IAI.00268-13 23798529 PMC3754193

[B4] Blume C. David J. Bell R. E. Laver J. R. Read R. C. Clark G. C. (2016). Modulation of human airway barrier functions during *Burkholderia thailandensis* and *Francisella tularensis* infection running title: airway barrier functions during bacterial infections. *Pathogens* 5:E53 10.3390/pathogens5030053 PMC5039433 27527221

[B5] Borosmajewska J. Wei X. Milewski S. Williams D. W. (2015). A novel in vitro assay for assessing efficacy and toxicity of antifungals using human leukemic cells infected with *Candida albicans*. *J. Appl. Microbiol.* 119 177–187. 10.1111/jam.12817 25845720

[B6] Bruewer M. Samarin S. Nusrat A. (2006). Inflammatory bowel disease and the apical junctional complex. *Ann. N. Y. Acad. Sci.* 1072 242–252. 10.1196/annals.1326.017 17057204

[B7] Burns J. L. Griffith A. Barry J. J. Jonas M. Chi E. Y. (2001). Transcytosis of gastrointestinal epithelial cells by *Escherichia coli* K1. *Pediatr. Res.* 49 30–37. 10.1203/00006450-200101000-00010 11134488

[B8] Cammarota G. Ianiro G. Bibbò S. Gasbarrini A. (2014). Gut microbiota modulation: probiotics, antibiotics or fecal microbiota transplantation? *Intern. Emerg. Med.* 9 365–373. 10.1007/s11739-014-1069-4 24664520

[B9] Cilieborg M. S. Thymann T. Siggers R. Boye M. Bering S. B. Jensen B. B. (2011). The incidence of necrotizing enterocolitis is increased following probiotic administration to preterm pigs. *J. Nutr.* 141 223–230. 10.3945/jn.110.128561 21178092

[B10] Claud E. C. (2009). Neonatal necrotizing enterocolitis –inflammation and intestinal immaturity. *Antiinflamm. Antiallergy Agents Med. Chem.* 8 248–259. 10.2174/187152309789152020 20498729 PMC2874244

[B11] Constantinovits M. Sipos F. Molnár B. Tulassay Z. Mûzes G. (2012). Organizer and regulatory role of colonic isolated lymphoid follicles in inflammation. *Acta Physiol. Hung.* 99 344–352. 10.1556/APhysiol.99.2012.3.11 22982722

[B12] Corfield A. P. Carroll D. Myerscough N. Probert C. S. (2001). Mucins in the gastrointestinal tract in health and disease. *Front. Biosci.* 6 D1321–D1357. 11578958 10.2741/corfield

[B13] Deshmukh H. S. Liu Y. Menkiti O. R. Mei J. Dai N. O’leary C. E. (2014). The microbiota regulates neutrophil homeostasis and host resistance to *Escherichia coli* K1 sepsis in neonatal mice. *Nat. Med.* 20 524–530. 10.1038/nm.3542 24747744 PMC4016187

[B14] Fransen F. Zagato E. Mazzini E. Fosso B. Manzari C. El A. S. (2015). BALB/c and C57BL/6 mice differ in polyreactive IgA abundance, which impacts the generation of antigen-specific IgA and microbiota diversity. *Immunity* 43 527–540. 10.1016/j.immuni.2015.08.011 26362264

[B15] Fukuda S. Toh H. Hase K. Oshima K. Nakanishi Y. Yoshimura K. (2011). Bifidobacteria can protect from enteropathogenic infection through production of acetate. *Nature* 469 543–547. 10.1038/nature09646 21270894

[B16] Furuya E. Y. Lowy F. D. (2006). Antimicrobial-resistant bacteria in the community setting. *Nat. Rev. Microbiol.* 4 36–45. 10.1038/nrmicro1325 16357859

[B17] Garcia M. A. Yang N. Quinton P. M. (2009). Normal mouse intestinal mucus release requires cystic fibrosis transmembrane regulator-dependent bicarbonate secretion. *J. Clin. Investig.* 119 3497–3497. 10.1172/JCI38662 PMC2735925 19726884

[B18] Glode M. P. Sutton A. Moxon E. R. Robbins J. B. (1977). Pathogenesis of neonatal *Escherichia coli* meningitis: induction of bacteremia and meningitis in infant rats fed *E. coli* K1. *Infect. Immun.* 16 75–80. 326679 10.1128/iai.16.1.75-80.1977 PMC421490

[B19] Hansson G. C. (2012). Role of mucus layers in gut infection and inflammation. *Curr. Opin. Microbiol.* 15 57–62. 10.1016/j.mib.2011.11.002 22177113 PMC3716454

[B20] Holt D. E. Halket S. Louvois J. D. Harvey D. (2001). Neonatal meningitis in England and Wales: 10 years on. *Arch. Dis. Child. Fetal Neonatal Ed.* 84 F85–F89. 10.1136/fn.84.2.F85 11207221 PMC1721232

[B21] Jakaitis B. M. Denning P. W. (2014). Commensal and probiotic bacteria may prevent NEC by maturing intestinal host defenses. *Pathophysiology* 21 47–54. 10.1016/j.pathophys.2013.11.012 24440614 PMC5424473

[B22] Kim K. S. (2003). Pathogenesis of bacterial meningitis: from bacteraemia to neuronal injury. *Nat. Rev. Neurosci.* 4 376–385. 10.1038/nrn1103 12728265

[B23] Kim Y. S. Ho S. B. (2010). Intestinal goblet cells and mucins in health and disease: recent insights and progress. *Curr. Gastroenterol. Rep.* 12 319–330. 10.1007/s11894-010-0131-2 20703838 PMC2933006

[B24] Koutsounas I. Kaltsa G. Siakavellas S. I. Bamias G. (2015). Markers of bacterial translocation in end-stage liver disease. *World J. Hepatol.* 7 2264–2273. 10.4254/wjh.v7.i20.2264 26380651 PMC4568487

[B25] Kronman M. P. Zaoutis T. E. Haynes K. Feng R. Coffin S. E. (2012). Antibiotic exposure and IBD development among children: a population-based cohort study. *Pediatrics* 130 e794–e803. 10.1542/peds.2011-3886 23008454 PMC4074626

[B26] Li X. Q. Zhu Y. H. Zhang H. F. Yue Y. Cai Z. X. Lu Q. P. (2012). Risks associated with high-dose *Lactobacillus rhamnosus* in an *Escherichia coli* model of piglet diarrhoea: intestinal microbiota and immune imbalances. *PLOS ONE* 7:e40666 10.1371/journal.pone.0040666 PMC3407149 22848393

[B27] Macpherson A. J. Mccoy K. D. Johansen F. E. Brandtzaeg P. (2008). The immune geography of IgA induction and function. *Mucosal Immunol.* 1 11–22. 10.1038/mi.2007.6 19079156

[B28] Madan J. C. Salari R. C. Saxena D. Davidson L. O’toole G. A. Moore J. H. (2012). Gut microbial colonisation in premature neonates predicts neonatal sepsis. *Arch. Dis. Child. Fetal Neonatal Ed.* 97 456–462. 10.1136/fetalneonatal-2011-301373 PMC3724360 22562869

[B29] Naydenov N. G. Ivanov A. I. (2010). Adducins regulate remodeling of apical junctions in human epithelial cells. *Mol. Biol. Cell* 21 3506–3517. 10.1091/mbc.E10-03-0259 20810786 PMC2954116

[B30] Nie C. Zhou J. Qin X. Shi X. Zeng Q. Liu J. (2016). Reduction of apoptosis by proanthocyanidin-induced autophagy in the human gastric cancer cell line MGC-803. *Oncol. Rep.* 35 649–658. 10.3892/or.2015.4419 26572257 PMC4689485

[B31] Ouwerkerk J. P. De Vos W. M. Belzer C. (2013). Glycobiome: bacteria and mucus at the epithelial interface. *Best Pract. Res. Clin. Gastroenterol.* 27 25–38. 10.1016/j.bpg.2013.03.001 23768550

[B32] Patel R. M. Myers L. S. Kurundkar A. R. Maheshwari A. Nusrat A. Lin P. W. (2012). Probiotic bacteria induce maturation of intestinal claudin 3 expression and barrier function. *Am. J. Pathol.* 180 626–635. 10.1016/j.ajpath.2011.10.025 22155109 PMC3349863

[B33] Pearson C. Uhlig H. H. Powrie F. (2012). Lymphoid microenvironments and innate lymphoid cells in the gut. *Trends Immunol.* 33 289–296. 10.1016/j.it.2012.04.004 22578693

[B34] Pietzak M. M. Chakraborty E. Wang Y. Huang S. (2004). P0173 PP Deletion of s-fimbriae operon abrogates *E. coli* K1 induced apoptosis in intestinal epithelial cells. *J. Pediatr. Gastroenterol. Nutr.* 39 S124. 10.1097/00005176-200406001-00297

[B35] Rousset M. (1986). The human colon carcinoma cell lines HT-29 and Caco-2: two in vitro models for the study of intestinal differentiation. *Biochimie* 68 1035–1040. 10.1016/S0300-9084(86)80177-8 3096381

[B36] Servin A. L. (2004). Antagonistic activities of lactobacilli and bifidobacteria against microbial pathogens. *FEMS Microbiol. Rev.* 28 405–440. 10.1016/j.femsre.2004.01.003 15374659

[B37] Simonsen K. A. Andersonberry A. L. Delair S. F. Davies H. D. (2014). Early-onset neonatal sepsis. *Clin. Microbiol. Rev.* 27 21–47. 10.1128/CMR.00031-13 24396135 PMC3910904

[B38] Smith K. Mccoy K. D. Macpherson A. J. (2007). Use of axenic animals in studying the adaptation of mammals to their commensal intestinal microbiota. *Semin. Immunol.* 19 59–69. 10.1016/j.smim.2006.10.002 17118672

[B39] Soman N. R. Marsh J. N. Lanza G. M. Wickline S. A. (2008). New mechanisms for non-porative ultrasound stimulation of cargo delivery to cell cytosol with targeted perfluorocarbon nanoparticles. *Nanotechnology* 19:185102 10.1088/0957-4484/19/18/185102 PMC3074498 21494419

[B40] Sommer F. Bäckhed F. (2013). The gut microbiota-masters of host development and physiology. *Nat. Rev. Microbiol.* 11 227–238. 10.1038/nrmicro2974 23435359

[B41] Soriano G. Sánchez E. Guarner C. Schiffrin E. J. (2012). *Lactobacillus johnsonii* La1 without antioxidants does not decrease bacterial translocation in rats with carbon tetrachloride-induced cirrhosis. *J. Hepatol.* 57 1395–1396. 10.1016/j.jhep.2012.07.019 22824820

[B42] Tsilingiri K. Barbosa T. Penna G. Caprioli F. Sonzogni A. Viale G. (2012). Probiotic and postbiotic activity in health and disease: comparison on a novel polarised ex-vivo organ culture model. *Gut* 61 1007–1015. 10.1136/gutjnl-2011-300971 22301383

[B43] Tsilingiri K. Rescigno M. (2012). Postbiotics: What else? *Benef. Microbes* 4 101–107. 10.3920/BM2012.0046 23271068

[B44] Valeri M. Paccani S. R. Kasendra M. Nesta B. Serino L. Pizza M. (2015). Pathogenic *E. coli* exploits SslE mucinase activity to translocate through the mucosal barrier and get access to host cells. *PLOS ONE* 10:e0117486 10.1371/journal.pone.0117486 PMC4366376 25789808

[B45] Wang S. Peng L. Gai Z. Zhang L. Jong A. Cao H. (2016). Pathogenic triad in bacterial meningitis: pathogen invasion, NF-κB activation, and leukocyte transmigration that occur at the blood-brain barrier. *Front. Microbiol.* 7:148 10.3389/fmicb.2016.00148 PMC4760054 26925035

[B46] Watson C. L. Mahe M. M. Múnera J. Howell J. C. Sundaram N. Poling H. M. (2014). An in vivo model of human small intestine using pluripotent stem cells. *Nat. Med.* 20 1310–1314. 10.1038/nm.3737 25326803 PMC4408376

[B47] Weaver L. T. Laker M. F. Nelson R. (1984). Intestinal permeability in the newborn. *Arch. Dis. Child.* 59 236–241. 10.1136/adc.59.3.236 6424583 PMC1628529

[B48] Weiser J. N. Gotschlich E. C. (1991). Outer membrane protein A (OmpA) contributes to serum resistance and pathogenicity of *Escherichia coli* K-1. *Infect. Immun.* 59 2252–2258. 1646768 10.1128/iai.59.7.2252-2258.1991 PMC258003

[B49] Witcomb L. A. Collins J. W. Mccarthy A. J. Frankel G. Taylor P. W. (2015). Bioluminescent imaging reveals novel patterns of colonization and invasion in systemic *Escherichia coli* K1 experimental infection in the neonatal rat. *Infect. Immun.* 83 4528–4540. 10.1128/IAI.00953-15 26351276 PMC4645386

[B50] Wittig B. Zeitz M. (2003). The gut as an organ of immunology. *Int. J. Colorectal Dis.* 18 181–187. 12673481 10.1007/s00384-002-0444-1

[B51] Yan F. Liu L. Cao H. Moore D. J. Washington M. K. Wang B. (2016). Neonatal colonization of mice with LGG promotes intestinal development and decreases susceptibility to colitis in adulthood. *Mucosal Immunol.* 10 117–127. 10.1038/mi.2016.43 27095077 PMC5073052

[B52] Yang F. Wang A. Zeng X. Hou C. Liu H. Qiao S. (2015). *Lactobacillus reuteri* I5007 modulates tight junction protein expression in IPEC-J2 cells with LPS stimulation and in newborn piglets under normal conditions. *BMC Microbiol.* 15:32. 10.1186/s12866-015-0372-1 PMC4350629 25888437

[B53] Yousuf F. A. Yousuf Z. Iqbal J. Siddiqui R. Khan H. Khan N. A. (2014). Interactions of neuropathogenic *Escherichia coli* K1 (RS218) and its derivatives lacking genomic islands with phagocytic acanthamoeba castellanii and nonphagocytic brain endothelial cells. *Biomed Res. Int.* 2014:265424 10.1155/2014/265424 PMC4004053 24818136

[B54] Yu J. Y. He X. L. Puthiyakunnon S. Peng L. Li Y. Wu L. S. (2015). Mucin2 is required for probiotic agents-mediated blocking effects on meningitic *E. coli*-induced pathogenicities. *J. Microbiol. Biotechnol.* 25 1751–1760. 10.4014/jmb.1502.02010 26059517

[B55] Zareie M. Johnson-Henry K. Jury J. Yang P. C. Ngan B. Y. Mckay D. M. (2006). Probiotics prevent bacterial translocation and improve intestinal barrier function in rats following chronic psychological stress. *Gut* 55 1553–1560. 10.1136/gut.2005.080739 16638791 PMC1860130

